# A Familiar Working Environment Influences Surgeon’s Stress in the Operating Room: A Multi-Specialty Prospective Cohort Study

**DOI:** 10.1097/SLA.0000000000006543

**Published:** 2024-10-01

**Authors:** Jake A. Awtry, Sarah C. Skinner, Léa Pascal, Stephanie Polazzi, Jean-Christophe Lifante, Antoine Duclos, TopSurgeons Study Group, Jake A Awtry

**Affiliations:** * Center for Surgery and Public Health, Department of Surgery, Brigham and Women’s Hospital, Harvard Medical School, Boston, MA; † Division of Cardiac Surgery, Brigham and Women’s Hospital, Boston, MA; ‡ Research on Healthcare Performance RESHAPE, Inserm U1290, Université Claude Bernard Lyon 1, Lyon, France; § Department of Endocrine Surgery, Lyon Sud Hospital, Hospices Civil de Lyon, Lyon, France

**Keywords:** heart rate variability, human factors, operating room environment, operating room familiarity, stress, team familiarity, vagal tone

## Abstract

**Objective:**

To determine the influence of operating room familiarity on surgeon stress.

**Background:**

Regulating surgeon stress may improve patient safety. This study evaluated how assisting surgeons and operating room familiarity influence intraoperative heart rate variability among surgeons.

**Methods:**

Attending surgeons from 7 specialties within 4 university hospitals in France were enrolled from November 1, 2020 to December 31, 2021. Vagal tone, an indicator of stress derived from heart rate variability, was assessed during the first 5 minutes after incision using the root mean square of successive differences (RMSSD). Higher RMSSD values indicate greater vagal tone. Team familiarity was quantified as the cumulative time the attending and assisting surgeons had operated together in the past, while operating rooms in which the surgeon conducted >10% of their operations were termed familiar. The effect of each on the RMSSD was assessed via a linear mixed-effect model adjusting for the random effect of the surgeon and possible confounders.

**Results:**

Overall, 643 surgeries performed by 37 surgeons were included. The median surgeon age was 49 years; 299 (78.4%) were male, and 22 (59.5%) were professors. Surgeons spent an average of 21.2 hours with the assisting surgeon before surgery and conducted 585 (91.0%) of their operations in a familiar operating room. For every 10 additional hours spent operating together, ln(RMSSD) significantly increased by 0.018 (95% CI: 0.003 to 0.033, *P*=0.016). Familiar operating rooms also tended to increase surgeon ln(RMSSD) [0.098 (95%CI: −0.007 to 0.203, *P*=0.068)].

**Conclusion:**

Familiar with assisting surgeons and potentially operating rooms, increased surgeon vagal tone. Maintaining a stable operating room environment may improve surgeon stress and patient care.

The operating room is a dynamic, interdisciplinary setting in which collaborative decision-making and performance have significant consequences for patients. Traditional efforts to elicit favorable outcomes have emphasized patient and surgical technique optimization. However, it is increasingly recognized that nontechnical skills for surgeons such as team leadership, communication, situational awareness, and behavioral regulation are meaningful contributors to operating room team dynamics and effectiveness.^1–3^ Therefore, focusing on elements of the surgeon themself that can be concertedly modulated offers a potentially novel alternative to bettering patient outcomes. Collectively, these concepts are encapsulated in surgical human factors research, which recognizes the role that systems and culture play in modifying the experiences, characteristics, and capabilities of surgeons.^4^

One such human factor element known to impact surgical performance is stress. The relationship between stress and performance is complex,^5,6^ but excessive surgeon stress or cognitive overload can both contribute to surgical error^7,8^ and compromise surgeon well-being.^9^ Stress can be acute or chronic in nature, engendered by factors both extrinsic and intrinsic to the operating room including lifestyle, intraoperative complications, and unexpected events like equipment failures.^10,11^ Structural aspects of the surgical profession contribute to chronic stress^9,10^ and may be challenging to ameliorate, though acute stress has been associated with surgeon technical^8^ and nontechnical^7^ skills and is potentially more readily manipulated. While acute surgeon stress has traditionally been examined via retrospective surveys, these are difficult to administer in the operating room and prone to bias.^12,13^ Alternatively, acute stress can be objectively quantified in real-time by physiological measures, most commonly via changes in heart rate variability (HRV).^14,15^ While multiple distinct metrics of HRV have been employed, the root mean square of successive differences (RMSSD) in interbeat intervals correlates strongly with autonomic vagal tone and is less subject to interference from other physiological perturbations such as respiration.^16,17^ Importantly, vagal tone specifically has been shown to improve stress response and cognitive functioning.^17,18^ Therefore, RMSSD provides a useful mechanism to examine influences on a physiological metric of vagal tone and stress that may have significant implications for surgeon ability.

Another human factors element recognized to be important in surgery is team familiarity, which quantifies the extent of collaboration between members of the surgical team over time. Previously, increased team familiarity between the primary surgeon and other surgical and anesthesiology team members has been associated with increased efficiency^19–21^ and reduced postoperative complications.^22,23^ Despite these observations, the mechanism of team performance improvement with increased familiarity and the direct effect on the surgeon remains opaque. In addition, other contributors to familiar operating environments, such as the operating room itself, have not been previously considered. Clarifying how a stable operating room environment affects surgeons at the start of surgery may aid future efforts to leverage it clinically.

The present study aimed to deepen our understanding of the role of human factors considerations in surgery by examining influences on surgeon vagal tone and stress. Specifically, we interrogated how attending surgeon RMSSD in the first 5 minutes after incision varies in response to familiarity with the surgical assistant and the physical operating room, hypothesizing that increased familiarity significantly contributes to increased vagal tone, ergo reduced acute stress. The focus on the beginning of surgery emphasizes the baseline physiological state of the surgeon before the influence of intraoperative events, such as unanticipated patient complications or organizational disruptions, which may expectedly elicit adaptive autonomic system changes. In doing so, we sought to provide a link between operating room systems characteristics and surgeon physiological stress that may, in part, explain variations in performance.

## METHODS

### Study Design and Population

This multi-institutional prospective observational cohort study included 37 attending surgeons (annual surgical volume ≥50 cases) across 14 surgical departments from four university hospitals in France. Surgeons represented 7 specialties including digestive, orthopedic, gynecologic, urologic, cardiac, thoracic, and endocrine surgery. Intraoperative heart rate variability data were collected during 5 regularly spaced 15-day sessions between November 1, 2020 and December 31, 2021. Exclusion criteria included patients younger than 18 years old or who refused to share their personal data, operations for palliative care or organ donation, surgeries performed at night, and operations missing event timestamping. In addition, operations performed by a single attending surgeon without an assisting surgeon were not analyzed.

The study used pseudonymized data in accordance with European General Data Protection Regulation No. 2016/679. It was approved by the French National Data Protection Authority (DR-2020-055 CNIL) and the European Research Council Executive Agency (801660 ERCEA). It was deemed exempt from formal oversight by the Mass General Brigham Institutional Review Board (Protocol 2023P002266). Patients were informed that their health data might be reused for research purposes and given the opportunity to opt out. Each surgeon provided informed consent to participate in the research and for the use of their data.

### Data Sources and Exposure Measurement

For each operation, data were prospectively collected from a homogeneous information system across the Lyon University hospitals, encompassing details regarding the operation, patient, surgeon, and operating room involved. Specifically, we gathered data on the patients’ sociodemographic characteristics, care provided during hospital stays, details about the type of surgery performed (organ, surgical approach, and complexity), and the primary diagnosis linked to the operative indication.

We supplemented this information with data collected by clinical research assistants from the patient’s electronic health records to acquire details such as the scheduling of the operation (elective, semi-urgent, urgent), the preoperative comorbidities of the patient, as well as the type of anesthesia administered and the patients’ ASA Physical Status Classification System. In addition, for each operation, we used data from the operating room management software and the surgical procedure report to determine the timings of the patient’s entry into the operating room, the incision and closure of the wound, and the attending and assisting surgeons primarily involved. Lastly, we gathered human resources data to determine the age and professional status (professor vs nonprofessor) of the attending surgeon.

The primary exposures were (1) the surgical familiarity between the attending and assisting surgeons, as quantified by the number of hours spent operating together during the study up to the date of surgery, and (2) the familiarity of the operating room to the attending surgeon, with operating rooms used during >10% of a given surgeon’s case volume termed familiar. Given the arbitrary nature of the cut-off used to define familiar operating rooms, a sensitivity analysis was conducted defining familiar operating rooms as those constituting >20% of a surgeon’s case volume to test the robustness of our results.

### Heart Rate Variability Data Collection

During each surgery, attending surgeons wore ActiGraph GT3X-BT (ActiGraph, Pensacola, FL) ankle monitors to assess motion and Polar H10 chest belts (Polar Electro, Kempele, Finland) to measure heart rate interbeat intervals for a 5-minute period, as previously recommended to ensure the accuracy of short-term heart rate variability assessments.^24^ The focus on the beginning of the operation limits confounding from potential stressors that may arise during surgery. The collected data were validated, corrected for noise, and analyzed using Kubios HRV 4.0 software. Data was excluded for patients with operations <20 minutes, for surgeons who had no Polar data recorded for ≥5 minutes, and for surgeons with ≥5% beat correction. Because heart rate variability is impacted by the effect of physical activity on sympathetic and vagal tone,^25^ data was also excluded for surgeons who had no period of immobility ≥20 minutes. Similar to previous studies examining vagal tone, surgeon vagal tone was quantified via the RMSSD.^26^ A higher RMSSD indicates higher vagal tone and lower stress.

### Statistical Analysis

Patient, surgeon, and surgery characteristics were presented as mean and SD for normally distributed continuous variables, median [interquartile range] (IQR) for non-normally distributed variables, and frequencies with percentages for categorical variables. The adjusted associations between the exposures of interest and surgeon vagal tone were assessed via a linear mixed-model incorporating the random effect of the surgeon to account for clustering of surgeries within each surgeon. To control for confounding, the model included a preoperative risk score calculated for each surgery based on a separate data set from the same cohort of surgeons between January 1, 2022 and October 31, 2022 (Appendix 1 for details, Supplemental Digital Content 1, http://links.lww.com/SLA/F315); surgeon age, sex, professional status, and operative time during the study period (high vs low) dichotomized based on the population median; and the time of incision expressed as hours after midnight (due circadian variations in vagal tone^27^). Results of the multivariable model were displayed as the adjusted coefficient estimates (β) with 95% CI and also depicted graphically over a range of surgeon-assistant familiarity. To quantify the association between the exposures of interest and vagal tone, we applied a natural logarithmic transformation to RMSSD [ln(RMSSD)] due to the non-normal distribution of RMSSD in our study population. For all analyses, a 2-sided *P* value <0.05 defined statistical significance. Data manipulation and analyses were conducted using SAS V.9.4 (SAS Institute, Cary, NC).

## RESULTS

A total of 37 surgeons were included in the study. Median [interquartile range] surgeon age was 49,^13^ 29 (78.4)% were male, and 22 (59.5%) were either associate or full professors. Median cumulative operating time during the study period was 22,039 [13,647, 28,073] minutes. The median RMSSD for the primary surgeon during the first five minutes of surgery was 17.0 [10.8, 24.7] ms (Table 1), representing decreased vagal tone relative to the population-average resting RMSSD in individuals of a similar age (30.0 ms for people aged 45–54 years).^28^

**TABLE 1:** Patient and Surgery Characteristics (N=643)

Patient characteristics	
Age of patient in years, mean (SD)	57.8 (16.4)
Female patient, N (%)	339 (52.7)
ASA classification, mean (SD)	2.1 (0.8)
Number of comorbidities*, mean (SD)	2.6 (2.2)
Risk score (missing=6), mean (SD)	0.2 (0.2)
Surgery characteristics	
Specialty, N (%)	
Cardiac	74 (11.5)
* *Endocrine	72 (11.2)
* *Digestive	127 (19.8)
* *Gynecologic	118 (18.4)
* *Orthopedic	149 (23.2)
* *Thoracic	25 (3.9)
* *Urologic	78 (12.1)
Principal anesthesia technique, N(%)	
General	539 (83.8)
Locoregional	104 (16.2)
Surgical operative time in minutes between incision and closure of the wound, mean (SD)	111.9 (89.0)
Hour of incision time, mean (SD)	11:48 (2:34)
Scheduling of the operation (missing=2), N (%)	
Elective	599 (93.4)
Semiurgent	5 (0.8)
Urgent	37 (5.8)
Initial surgical approach (missing=3), N (%)	
Open	414 (64.7)
Robotic-assisted	30 (4.7)
Videoscopic	152 (23.8)
Endoscopic	44 (6.9)
Surgeon characteristics	
Age (y), median [IQR]	49 [42, 55]
Male, N (%)	29 (78.4)
Associate or full professor, N(%)	22 (59.5)
RMSSD the first 5 min after incision, median [IQR]	17.0 [10.8, 24.7]
Team familiarity–cumulative operating time with the assisting surgeon (h), mean (SD)	21.2 (30.4)

* See Supplemental Table 1, Supplemental Digital Content 1, http://links.lww.com/SLA/F315 for frequencies of all patient comorbidities in the cohort.

ASA indicates American Society of Anesthesiologist; IQR, interquartile range.

**FIGURE 1:**
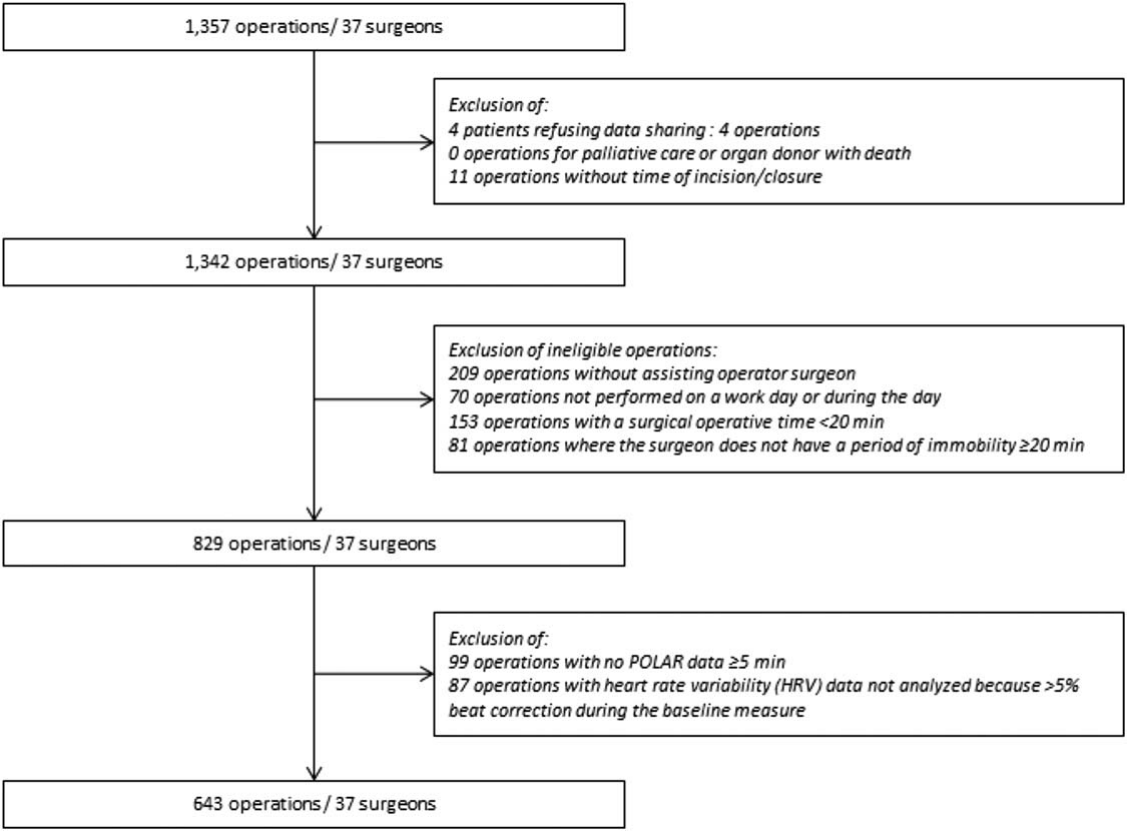
Flowchart of patient inclusion and exclusion criteria. The flowchart indicates the included and excluded cases, culminating in the final cohort of 643 cases. Exclusion criteria were applied to optimize the reliability of heart rate variability recordings.

**FIGURE 2:**
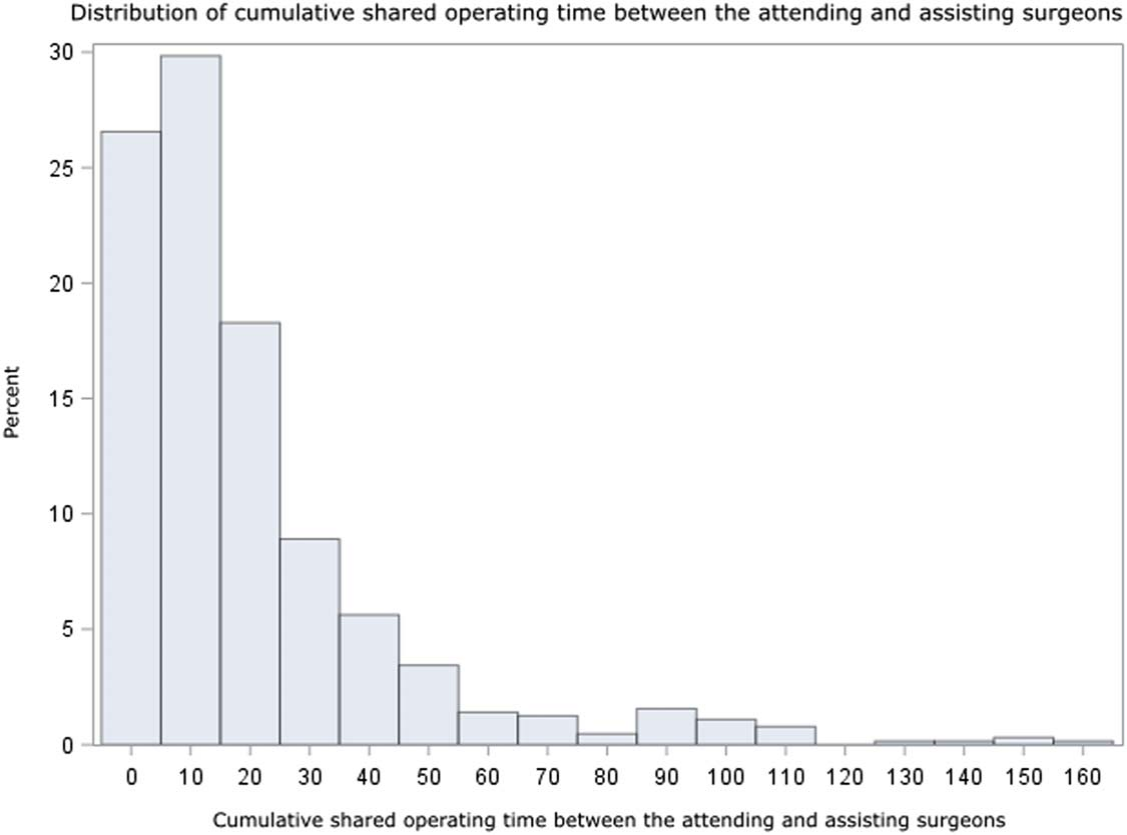
Distribution of team familiarity. Histogram of cumulative shared operating time during the study period between the attending and assisting surgeons.

After applying exclusion criteria, 643 surgeries were included (Fig. 1). The mean (SD) patient age was 57.8 (16.4) years old, 339 (52.7%) were female, and they had 2.6 (2.2) comorbidities (Table 1). The most common comorbidities included hypertension (35.9%), obesity (23.0%), cancer (21.3%), tobacco addiction (18.4%), and cardiovascular disease (16.3%) (Supplemental Table 1, Supplemental Digital Content 1, http://links.lww.com/SLA/F315). Surgeries were predominantly elective (599, 93.4%) and performed under general anesthesia (539, 83.8%) via an open approach (414, 64.7%). The surgical specialties represented included orthopedic (149, 23.2%), digestive (127, 19.8%), gynecologic (118, 18.4%), urologic (78, 12.1%), cardiac (74, 11.5%), endocrine (72, 11.2%), and thoracic (25, 3.9%) surgery. The operating room was familiar to the attending surgeon in 585 (91.0%) cases, and the attending had operated with the assisting surgeon for a mean of 21.2 (30.4) hours previously during the study period (Fig. 2). See Table 1 for further details.

**FIGURE 3:**
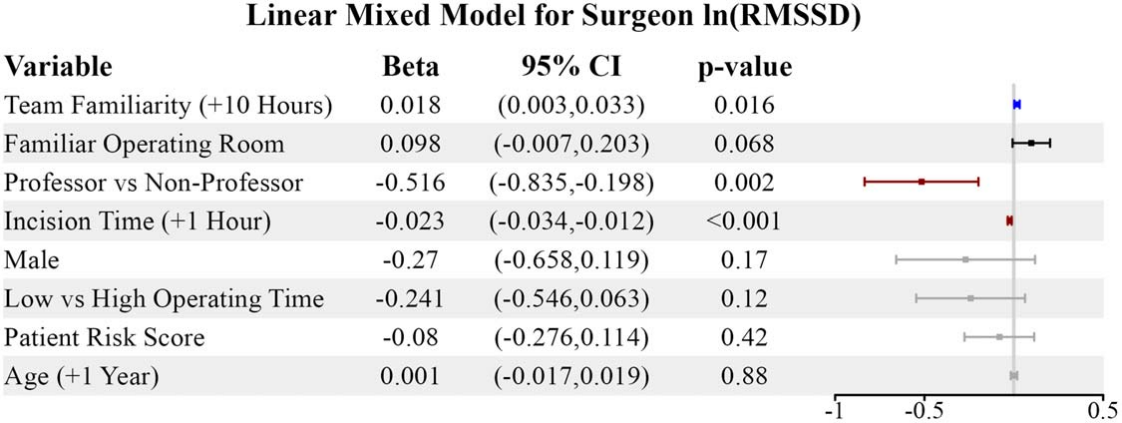
Linear mixed model for surgeon ln(RMSSD). A linear mixed model relating the exposures of interest to the natural logarithm of surgeon RMSSD with adjustment for potentially confounding surgeon, patient, and surgery characteristics. The forest plot on the right visually displays each predictor variable’s coefficient and 95% CI. Blue—significant increase in ln(RMSSD), *P*<0.05; black—marginally significant (0.05≤|*P*|<0.10); red—significant decrease in ln(RMSSD, *P*<0.05); gray—nonsignificant association with ln(RMSSD), *P*≥0.10.

**FIGURE 4:**
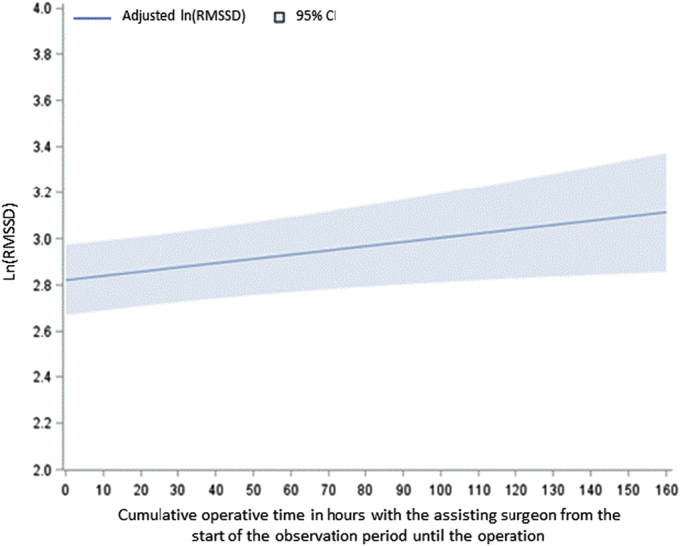
Relationship between team familiarity and surgeon vagal tone. Graphical depiction of the dose-response relationship between team familiarity and attending surgeon vagal tone, using beta coefficients, as estimated from the linear mixed model in Figure 3, with each confounder set to its mean value.

After adjustment for potential confounders, we observed a significant positive relationship between ln(RMSSD) and surgical team familiarity [β=0.018 (0.003 to 0.033), *P*=0.016] (Fig. 3). This relationship is depicted in Figure 4. Other predictors significantly associated with attending surgeon vagal tone included professor status and the time of incision. See Figure 3 for full details. Familiar operating rooms (>10% of a surgeon’s case volume) also tended to increase vagal tone, though this was marginally significant [β=0.098 (−0.007 to 0.203), *P*=0.068]. In the sensitivity analysis alternatively defining familiar operating rooms as those involved in >20% of a surgeon’s case volume, there was a statistically significant positive relationship between operating room familiarity and surgeon vagal tone [β=0.126 (0.048 to 0.205), *P*=0.002] (Supplemental Table 2, Supplemental Digital Content 1, http://links.lww.com/SLA/F315).

## DISCUSSION

This prospective, multi-institutional cohort study investigated how the surgical environment affects surgeon vagal tone and revealed several important findings. After controlling for potentially confounding surgeon and patient characteristics, there was a significant positive relationship between attending surgeon vagal tone and his or her familiarity with the assisting surgeon. A similar, though not statistically significant, trend was observed for familiarity with the operating room itself, with commonly utilized operating rooms favoring increased vagal tone. These results identify systems-level variables that can exert a positive influence on surgeon physiological stress measures known to impact performance and may, in part, explain the beneficial effect of team familiarity observed in prior literature.

Identifying systematic influences on surgeon vagal tone is important because of its prior association with cognitive performance and surgical technical outcomes. In the field of psychology, mounting evidence suggests that vagal tone contributes to interconnected aspects of cognition including executive function driven by the prefrontal cortex,^18^ emotional regulation,^29^ performance on theory of mind assessments,^30^ and adaptive behavioral responses to environmental disruptions.^31^ These cognitive skills may bolster integrative thinking in response to clinical developments in the operating room as well as facilitate interpersonal abilities required for nontechnical skills and leadership. Empirical evidence supports the translation of these benefits to surgical technical performance—lower RMSSD, corresponding to lower vagal tone, was associated with an increased rate of technical errors identified on operating room black box audiovisual recordings^32^ and with periods of error during simulated robotic surgeries.^33^ Interventions to improve vagal tone, then, offer promise in contributing to enhanced surgeon technical performance, though future studies are needed to directly investigate this possibility.

Our demonstration of the relationship between surgeon vagal tone and team familiarity offers new insight into how this phenomenon may evoke improved team performance. Intuitively, consistent collaboration between operating room team members may lead to shared expectations, better understanding of individual preferences, and congruent mental models and goals that could smoothen each step of the operation. Correspondingly, team familiarity, especially between the primary surgeon and assistants, has primarily been associated with improvements in operating room efficiency such as reduced operating times,^19^ turnover times,^34^ and cardiopulmonary bypass times.^21^ More difficult to understand, though, is how these improved efficiencies translate into significantly improved patient outcomes, as has been observed specifically for familiarity between the surgeon and anesthesiologist during complex gastrointestinal^23^ and cardiac^22^ surgery. Other observational studies have found that familiar teams may better identify and respond to errors^35^ and facilitate improved surgeon focus.^36^ Our association between improved surgeon team familiarity and increased surgeon vagal tone, coupled with the known impact of vagal tone on discrete cognitive pathways related to behavioral regulation and adaptation to stress, suggests that team familiarity may physiologically prepare a surgeon to respond effectively to complexity and complications encountered in the operating room. As evidence supporting the benefits of team familiarity grows, understanding the mediating forces contributing to improved team performance will be essential when looking to leverage it as a clinical tool.

Beyond the importance of surgical team personnel, environmental contributions from the operating room have also been shown to significantly impact surgeons. To our knowledge, how familiar operating rooms contribute to surgeon performance or experience has not been directly studied previously. Our findings demonstrate a trend toward increased vagal tone in familiar operating rooms that remains robust across differing thresholds used to define familiar operating rooms. While novel, this result is perhaps unsurprising based on previous literature. Dedicated operating rooms in orthopedic trauma surgery, which likely indirectly lead to increased operating room familiarity, have been associated with faster operative times and reduced patient morbidity.^37,38^ More broadly, elements such as noise, light, spatial ergonomics, visual distractions, and variations in the physical operating room have been noted to influence surgeon performance, potentially to a similar degree as technical ability.^39,40^ In addition, outside of surgery, familiar physical environments have been associated with improved spatial memory^41^ and learning,^42^ both of which could play an essential role in operating room performance. Maintaining a consistent working environment may, therefore, alleviate the cognitive load needed to adapt to an unfamiliar setting, thereby limiting added stress and distraction.

Our study has a number of strengths that allow our results to be interpreted confidently. Its prospective, multi-institutional and multi-specialty design utilizing an objective measure of physiological, rather than surgeon-reported, stress culminates in a unique data set that reduces the risk of bias. Unlike many previous human factors studies that rely on simulated surgeries conducted by residents, our data was collected from attending surgeons during real-world surgeries for actual patients. Further, the use of a physiological measure to quantify stress provides a quantitative model system for future efforts geared toward reproducing our results or identifying the optimal level of surgeon vagal tone. In addition, we were able to control for a number of surgeon and environmental characteristics known to influence heart rate variability to limit confounding. Most importantly, our analysis focuses on the first 5 minutes of surgery, thereby increasing our confidence that the observed relationships are predicated on baseline surgeon mental preparation, environmental influences, and responses to the operating room and team characteristics rather than changes provoked by events in the operating room. Finally, while most prior work has considered team familiarity as a simple count of shared cases between team members as a proxy for shared experience, our granular data allows us to directly tabulate shared operating time. This method may provide a more accurate assessment of team familiarity, obviating over-estimation or under-estimation introduced by variations in case lengths.

Conversely, our study has several limitations to consider. First, cases were nearly all elective and involved surgeons and patients from a restricted geographic area, which may limit its generalizability. Second, although we were able to control for several individual surgeon characteristics that can alter heart rate variability, we did not adjust for relative physical fitness, recent physical activity, medications, or diet, all of which may contribute to residual confounding affecting our results. For our team familiarity metric, we did not have access to the data needed to assess collaborations between the attending and assisting surgeons before the study period, and therefore we may either under or overestimate familiarity for specific dyads. To mitigate this, the study was designed to coincide with the start of the semester when residents rotate surgical services and were therefore unlikely to have prior collaboration with the attendings surgeons. In addition, although the influences of vagal tone on performance can be surmised from prior literature, our study did not directly collect data to validate variation in surgeon performance based on vagal tone. Finally, the study does not directly examine patient clinical outcomes, though this remains an active area of investigation separately.

Overall, our findings suggest that stable operating room organization with consistency in team and environmental structures contributes to increased vagal tone in surgeons, which may support cognitive processes that lead to better individual and team performance. The results highlight that surgeon physiological stress, and ultimately performance, are partly dependent on the daily working environment and provide opportunities for targeted systems engineering. While this concept has been recognized for several decades,^43^ related clinical interventions often have honed in on elements of health records, team communication, or clinical protocols^44,45^ rather than considering the surgeon themself as a modifiable element of the system. More recently, efforts have been made to develop platforms for human factors interventions centered on the surgeon, including using real-time heart rate variability data to identify optimal times for interruptions^46^ or provide alerts based on fluctuations in team member cognitive workload.^47^ Such human factors interventions may be particularly appealing because of the allosteric benefits they can provide for surgeons; for example, beyond the potential benefit to patients, increased vagal tone is also associated with reduced burnout^48^ and reduced cardiovascular disease.^49^ Autonomic tone may also be intentionally modified through breathing exercises with biofeedback training,^50^ thus providing a potential mechanism for surgeons to combat the detriments of unfamiliar environments if recognized.

Finally, it is important to note that human factors interventions may differentially affect surgeons with distinct needs and strengths. For example, although not the primary focus of the study, we found significantly reduced vagal tone for surgeons who were associate or full professors. This trend warrants further dedicated investigation but may be related to the competing teaching, clinical, and research demands of professors culminating in increased stress. For this reason, professors may particularly benefit from increased team and operating familiarity. Fostering surgical team familiarity and a stable operating room environment could provide a targeted organizational management strategy to optimize the stress of the attending surgeon and impact performance for patients.

## CONCLUSION

Attending surgeon familiarity with the assisting surgeon was significantly associated with increased attending vagal tone at the start of surgery with a similar, though not statistically significant, trend observed for familiar operating rooms. The results importantly highlight surgical system characteristics capable of beneficially affecting surgeon physiological stress, and offer a potential mechanism by which operating room familiarity can impact patient care. Future efforts to manipulate surgeon vagal tone offer promise to benefit both patients and surgeons.

## Supplementary Material
