# Supplementary material for: A Familiar Working Environment Influences Surgeon’s Stress in the Operating Room: A Multi-Specialty Prospective Cohort Study
Source: Ann Surg. 2024 Oct 1;283(4):620–7. doi: 10.1097/SLA.0000000000006543 (PMC12978712; doi:10.1097/SLA.0000000000006543)
Supplement: Supplementary file 1 [file sla-283-620-s001.docx]

**dsSUPPLEMENTAL MATERIAL**

**Table of Contents:**

Appendix 1: Details of Composite Risk Score Development

Supplemental Table 1: Patient Comorbidity Frequencies

Supplemental Table 2: Linear Mixed Model for Surgeon ln(RMSSD) - Sensitivity Analysis Re-Defining Familiar Operating Rooms

**Appendix 1:** **Details of Composite Risk Score Development**

A preoperative risk score was developed to predict the probability of major surgical adverse event occurrence (severe complication, unplanned reoperation, transfer in intensive unit related to organ failure, inpatient death) during the initial surgery or within 30 days following the initial surgery for each operation performed by surgeons. The risk score was developed using an independent training dataset (a 50% random sample) of 3,644 operations performed by the same cohort of surgeons during a separate time period - from January 1, 2022 to October 31, 2022. The following variables were systematically considered for inclusion in the models:

- Surgical procedure (767 distinct types of procedures spanning various surgical specialties)
- Surgical indication of the operation based on the chapter of the ICD-10 codes
- Scheduling of the operation (urgent, semi-urgent, elective)
- Type of anesthesia (general, regional, local) and surgical approach (open, videoscopic, endoscopic, robot)
- Patient demographics (age, sex) and socio-economic status (individual precarious situation and median income of the municipality of residence in quartiles)
- ASA physical status classification system (from 1 to 5)
- Comorbidities including critical condition, current pregnancy, obesity BMI ≥ 30 kg/m², malnourishment, tobacco/alcohol or other drug addictions, open wound, surgical site infection, sepsis, endocarditis, cancer, neoadjuvant treatment, immune deficiency, coagulopathy, anticoagulant or antiaggregation treatment, blood transfusion, coma, limb paralysis, other neurological disorder, confusion, dementia, depression, cardiovascular disease, neurovascular disease, peripheral arterial disease, cardiac arrhythmia, chronic heart failure, hypertension, diabetes, dyslipidemia, pulmonary artery systolic pressure > 60 mmHg, chronic renal failure, acute renal failure, chronic respiratory failure, chronic obstructive pulmonary disease, liver disease, rheumatic pathology, and hypoparathyroidism.

Considering all those potential confounders, models were subsequently trained using the operations from the independent dataset, grouping certain specialties due to low event rates as follows: cardiac with thoracic surgery, digestive with endocrine surgery, and orthopedic with urologic and gynecologic surgery. Variables were systematically selected using an automated stepwise logistic regression approach (entry threshold p = 0.20, exit threshold p = 0.10), enabling us to retain a specific set of variables for each specialties group. The obtained beta coefficients were then applied to the operations of the present observation study period (a 100% exhaustive sample of procedures performed by the cohort of surgeons, from November 1, 2020, to December 31, 2021), allowing us to estimate the probability of adverse event occurrence for each operation.

Models prediction performance was evaluated based on their calibration and discrimination with C-statistics and corresponding AUC on the implementation train dataset and on the observation test dataset for accuracy (external validation).

| Accuracy | Calibration | Discrimination |
| --- | --- | --- |
| Train : 86.5%  Test : 82.2% | 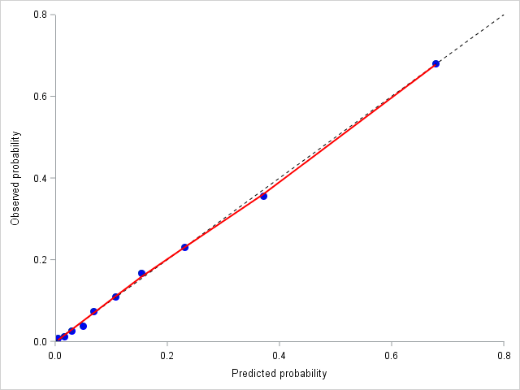 | 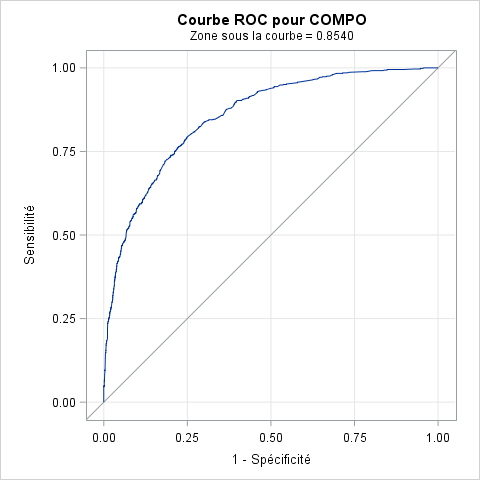  C-Stat=0.854 |

**Supplemental Table 1: Patient Comorbidity Frequencies**

| **Comorbidity** | **Frequency, N(%)** |
| --- | --- |
| Critical Condition | 5 (0.8) |
| Current Pregnancy | 2 (0.3) |
| Obesity | 148 (23.0) |
| Malnourishment | 10 (1.6) |
| Addiction |  |
| *Tobacco* | 118 (18.4) |
| *Alcohol* | 37 (5.8) |
| *Other* | 9 (1.4) |
| Open Wound | 0 (0.0) |
| Surgical Site Infection | 10 (1.6) |
| Sepsis | 9 (1.4) |
| Endocarditis | 2 (0.3) |
| Cancer | 137 (21.3) |
| Neoadjuvant Treatment | 37 (5.8) |
| Immune Deficiency | 39 (6.1) |
| Coagulopathy | 6 (0.9) |
| Anticoagulant Treatment | 69 (10.7) |
| Anti-Aggregation Treatment | 89 (13.8) |
| Blood Transfusion | 5 (0.8) |
| Coma | 0 (0.0) |
| Limb Paralysis | 2 (0.3) |
| Other Neurological Disorder | 23 (3.6) |
| Confusion | 4 (0.6) |
| Dementia | 5 (0.8) |
| Depression | 48 (7.5) |
| Cardiovascular Disease | 105 (16.3) |
| Neurovascular Disease | 27 (4.2) |
| Peripheral Arterial Disease | 19 (3.0) |
| Cardiac Arrhythmia | 52 (8.1) |
| Chronic Heart Failure | 12 (1.9) |
| Hypertension | 231 (35.9) |
| Diabetes | 84 (13.1) |
| Dyslipidemia | 106 (16.5) |
| High Pulmonary Artery Systolic Pressure | 3 (0.47) |
| Renal Failure |  |
| *Chronic* | 33 (5.1) |
| *Acute* | 9 (1.4) |
| Chronic Respiratory Failure | 5 (0.8) |
| Chronic Obstructive Pulmonary Disease | 27 (4.2) |
| Liver Disease | 13 (2.0) |
| Rheumatic Pathology | 104 (16.2) |
| Hypoparathyroidism | 14 (2.2) |

**Supplemental Table 2: Linear Mixed Model for Surgeon ln(RMSSD) - Sensitivity Analysis Re-Defining Familiar Operating Rooms**

| **Variable** | **Beta** | **95% CI** | **p-value** |
| --- | --- | --- | --- |
| Team Familiarity (+30 Hours) | 0.017 | [0.003,0.032] | **0.020** |
| Familiar Operating Room (>20% of Cases) | 0.126 | [0.048,0.205] | **0.002** |
| Professor vs Non-Professor | -0.524 | [-0.843,-0.204] | **0.001** |
| Incision Time (+1 Hour) | -0.023 | [-0.034,-0.012] | **<0.0001** |
| Male | -0.265 | [-0.655,0.125] | 0.18 |
| Low vs High Operating Time | -0.238 | [-0.544,0.068] | 0.13 |
| Patient Risk Score (+1 Score Unit) | -0.080 | [-0.274,0.114] | 0.42 |
| Age (+1 Year) | 0.001 | [-0.017,0.020] | 0.88 |

*Sensitivity analysis defining familiar operating rooms as those involved in >20% of a given surgeon’s case volume (Unfamiliar N = 110, Familiar N = 530). Linear mixed model relating the exposures of interest to the natural logarithm of surgeon RMSSD with adjustment for potentially confounding surgeon, patient, and surgery characteristics. P-values that meet the pre-specified criterion for significance (p<0.05) are bolded.*
